# ***ACS Central Science*** Virtual Issue on Advanced Materials and Processes for Building Low-Carbon Energy Systems

**DOI:** 10.1021/acscentsci.4c00925

**Published:** 2024-06-14

**Authors:** Chengyi Hu, Nanfeng Zheng

In the pursuit of a cleaner and more sustainable future, the global energy landscape is witnessing a transformative shift toward innovative technologies that address key challenges in energy security and environmental pollution.^1^This collection highlights the latest research on energy materials and technology innovations published in *ACS Central Science*. By including 44 representative research articles in this collection, we aim to provide a comprehensive overview of how advancements in energy production, storage, and conversion are driving the evolution toward a circular green economy (Figure 1).

**Figure 1:**
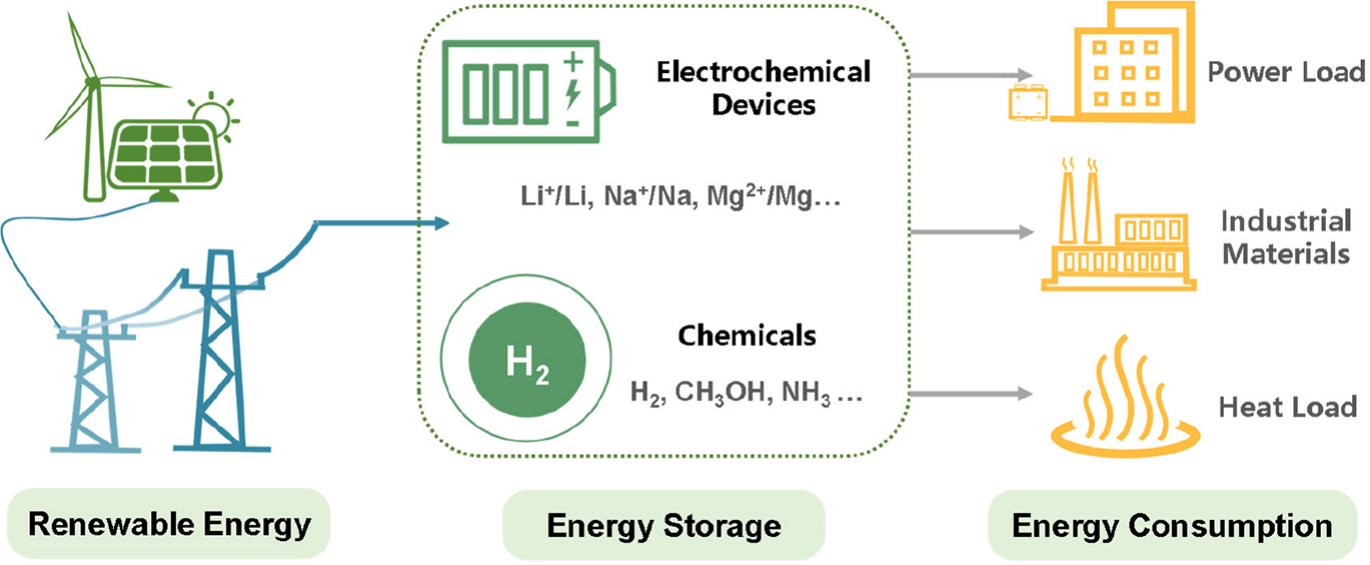
Collective research efforts toward a sustainable future through green energy technologies.

At the forefront of energy generation, perovskite solar cells have emerged as a transformative technology with profound implications for the photovoltaic industry. Characterized by exceptional light-absorption properties and cost-effectiveness in production, these cells hold immense promise for revolutionizing renewable energy systems. Significant research efforts have been directed toward enhancing the efficiency, stability, and scalability of perovskite solar cells, positioning them as viable candidates for widespread adoption in the sector of renewable energy production. Addressing the intermittency of renewable sources, energy storage assumes a pivotal role in ensuring grid stability and reliability. Advanced battery technologies have been instrumental in this regard, facilitating the efficient storage and retrieval of electrical energy. Through the integration of novel materials and design concepts, researchers have achieved notable progress in enhancing battery performance, extending cycle life, and accelerating charging rates. These advancements are essential for the realization of sustainable and reliable energy storage solutions. Furthermore, the reutilization of renewable energy sources, such as electrical and hydrogen energy, in diverse product applications presents a promising avenue for addressing both environmental concerns and energy challenges. By employing innovative energy conversion methodologies, we can optimize the integration and utilization of renewable energy resources, thereby advancing toward a more sustainable energy landscape.

## Materials for Renewable Electricity Generation

The development of metal-halide ABX_3_ perovskites as solar energy conversion materials has already led to single-junction perovskite solar cells (PSCs) with an impressive certified power conversion efficiency of 26.1%, receiving increasing attention in academia and industry. To further increase the efficiency of PSCs and thus outperform Si solar cells that are commonly used for solar electricity generation, a straightforward strategy is to create tandem PSCs by stacking bandgap-tunable perovskites together to harness the utilization of the solar spectrum and also minimize the thermalization losses. The global research effects have led to the development of all-perovskite tandem solar cells with an impressive efficiency of over 29%.

Tan and Saidaminov’s team envisions a roadmap for designing efficient and stable all-perovskite tandem solar cells.^2^ In their Outlook, together with a brief summary on the fundamentals and development of tandem PSCs, they discuss the scientific and engineering challenges associated with both absorbers and functional layers, including interconnection and charge-transport layers. Challenges such as phase segregation in the Br-rich wide-bandgap perovskite layer (∼1.8 eV) and oxidation-induced transformation of Sn^2+^ to Sn^4+^ in the mixed Pb–Sn narrow-bandgap perovskite layer (∼1.2 eV) were discussed. Strategies such as precursor composition, crystal growth control, and additive engineering were highlighted to enhance efficiency and stability. The discussion revolves around the triangle of efficiency, scalability, and stability, shedding light on remaining challenges and opportunities in tandem PSC development for practical applications.

Critical to the success of high-performance PSCs in competing with Si solar cells is the fabrication of large-area, high-quality halide perovskite films. While solvent engineering has emerged as an effective strategy for achieving high-efficiency PSCs, the use of high boiling point solvents such as *N*,*N*-dimethylformamide, dimethyl sulfoxide, and *N*-methylpyrrolidone in solution deposition processes is common. However, the challenge lies in the need for high-temperature annealing to obtain optimal efficiency, as excessive heat can lead to structural defects within perovskite films. These defects, acting as nonradiative carriers, adversely impact both efficiency and stability. Understanding the intricate role of different solvent molecules in perovskite film crystallization is crucial. Wu, Zheng, and their colleagues systematically investigated the solvent gaming chemistry during the crystallization of α-phase FAPbI_3_ perovskite, revealing the interplay among solvent basicity, solvent-contained intermediate structures, and the evolution from intermediate to perovskite.^3^ The identified solvent-gaming mechanism aids in guiding the preparation of intermediate structures, resulting in α-FAPbI_3_ with lower temperatures and high-quality perovskite films with minimized defects.

While substantial research endeavors have been devoted to advancing next-generation perovskite solar cells, a parallel avenue to collect and convert weak energy from nature has been explored. Taking inspiration from the hydrogel-based systems found in organisms, Wen and colleagues designed a hydrogel hybrid membrane for osmotic energy conversion. Their work introduces a space-charged hydrogel, markedly enhancing ion selectivity. When subjected to a salinity gradient, the hydrogel hybrid membrane-based osmotic power generator demonstrates remarkable efficiency in converting osmotic energy into electric power, underscoring the potential applications of membrane technology in energy conversion.^4^ In the work by Wang, Li et al., the sorption-induced reversible thermal effects were utilized to develop a near-zero-energy smart thermal management system for regulating battery temperature during heating and cooling processes, tailored to local conditions.^5^ This system employs a metal–organic framework composite material, MIL-101(Cr)@carbon foam, which utilizes automatic water vapor desorption/sorption to modulate battery temperature. This self-adaptive device effectively maintains battery temperature below 45 °C even during rapid charge/discharge cycles in hot climates, while it is also capable of self-heating to approximately 15 °C in cold environments. Kong et al. report an interfacial superassembly strategy to prepare a heterogeneous membrane of ordered mesoporous titania nanopillar-arrays/anodic aluminum oxide for modulating ion transport by light and pH stimulations.^6^ A light-responsive current density of 219.2 μA·cm^–2^ is achieved by the heterogeneous membrane.

## Materials for Lithium-Based Energy Storage Systems

The swift evolution of energy storage technologies is transforming industries globally, from consumer electronics to the integration of renewable energy sources. The articles showcased in this collection collectively highlight the remarkable progress made in the field of energy storage batteries. These studies explore groundbreaking developments in lithium-ion and sodium-ion battery technologies, along with inventive designs for electrodes and electrolytes, offering valuable insights into the future of energy storage. Additionally, the collection discusses the pivotal role of energy storage in propelling the shift toward clean energy sources, establishing smart grids, and effectively addressing pressing energy challenges. These advancements are crucial in facilitating the transition to a greener and more environmentally friendly energy future.

In an Outlook on lithium-ion battery (LIB) technology,^7^ Manthiram underscores the significance of LIBs in shaping our energy landscape. The insightful analysis in this Outlook dissects the prevalence of LIBs in various applications, meticulously weighing trade-offs between energy density, cycle life, cost, and environmental impact. While the existing technology relies on insertion-reaction electrodes and organic liquid electrolytes, the discussion also addresses the progress and challenges associated with next-generation LIBs. These advancements involve increased cell voltage, enhanced charge-storage capacity through novel electrode materials employing both insertion and conversion reactions, as well as the integration of solid electrolytes and lithium metal anodes. An Outlook by Zhang and colleagues addresses the challenges of developing practical Li–S batteries with high energy density.^8^ Key parameters including high-sulfur-loading cathodes, lean electrolyte, and limited anode excess are emphasized. They also highlight scientific problems such as low ionic conductivity causing notable ohmic polarization, saturation and premature precipitation of lithium polysulfides resulting in kinetic obstacles, and rapid degradation of Li metal anodes observed in Li–S pouch cells. The Outlook also proposes strategies to tackle these challenges.

As for new electrode materials, Yabuuchi et al. reported a high-energy cathode material by integrating nanostructured LiMnO_2_ with Li_3_PO_4_.^9^ Due to their different crystal structures, the uniform atom-scale mixing of LiMnO_2_ and Li_3_PO_4_ is impossible. The authors demonstrated that the dissolution of phosphorus ions into nanosized LiMnO_2_ resulted in a low-crystallinity nanostructure. In this metastable phase, phosphorus occupies tetrahedral sites and shares faces with adjacent lithium ions in distorted octahedral sites. The significant reversible capacity of ∼320 mA h g^–1^ exhibited by the developed material was attributed to the anionic redox of oxygen coupled with the cationic redox of Mn ions. The presence of phosphorus suppresses oxygen dimerization, contributing to improved reversibility in anionic redox. In another study, the team also explored the unexpected role of oxygen in charge compensation during the electrochemical oxidation of Ni-based materials, offering promise for designing advanced cathode materials with reversible anionic redox.^10^ Dincǎ and co-workers reported a metal-free LIB cathode material based on bis-tetraaminobenzoquinone (TAQ), a fused conjugated molecule having a layered packing structure in its solid form.^11^ The strong bonding and donor–acceptor π–π interactions among TAQ molecules make the solid material insoluble in electrolytes and electrically conductive. The TAQ cathode was demonstrated to have a specific capacity up to 306 mAh g^–1^ with an energy density of 765 Wh kg^–1^ and an excellent rate performance (full charging in 3 min).

To solve the capacity degradation problem of carbon anodes for LIBs operated at subzero temperatures, Wang, Yao, and co-workers prepared a carbon-based anode material possessing the Riemannian surface with a positive curvature. The anode exhibits a high reversible capacity of 624 mAh g^–1^ with more than 85% capacity retention at 0.1 A g^–1^ under −20 °C.^12^ They attributed the enhanced reversible capacities at low temperatures to the improved Li^+^ desolvation and charge transfer by the unique electronic structure of the as-built Riemannian surface. The development of lithium metal anodes is crucial to LIBs with high energy capacities. However, a persistent challenge remains in preventing dendrite growth during extreme fast charging for electric vehicles. To tackle the challenge, Lu, Feng, and He et al. developed an adaptive approach to suppress the Li dendrite formation by enhancing the internal electric field through constant voltage charging.^13^ The intensified electric field facilitated the migration and depletion of Li^+^ ions, resulting in a nitride-enriched solid electrolyte interphase (SEI) with NO_3_^–^ ions. The unique SEI layer played a crucial role in augmenting the overall stability and performance of the system.

In LIBs, electrolytes provide Li^+^-conducting media to deliver Li^+^ ions between cathodes and anodes during charging and discharging operations, and contact directly with cathodes, anodes, and also separators. Consequently, electrolytes play a pivotal role in regulating the performance of LIBs. In their Outlook, Srinivasan et al. suggest the establishment of a structure–property–performance relationship to quantitatively assess the relevance of each variation in Stefan-Maxwell diffusivity to electrolyte transport behavior within specific electrochemical contexts.^14^ In work by Wang, He, and co-workers, the influence of antisolvents on solvation structures and transport properties in localized high-concentration electrolytes was investigated.^15^ The investigation uncovered that antisolvents not only served as diluents for electrolytes but also induced low-dielectric environments and heightened inductive effects. Consequently, these factors altered the binding energies of Li^+^-solvent and Li^+^-anion interactions. Such effects significantly influence interfacial ion desolvation, Li^+^ transport, and thus the stability of anion reduction, underscoring the crucial role of antisolvents in advancing battery performance. Amanchukwu and colleagues developed a series of fluoroether electrolytes characterized by an ether moiety sandwiched between fluorinated end groups.^16^ These novel electrolytes exhibit impressive ionic conductivities, reaching up to 1.3 mS/cm at 30 °C with a 1 M salt concentration. Notably, their oxidative stability shows a correlation with the fluorine content, increasing as the fluorine content decreases.

The employment of liquid organic electrolytes in commercial LIBs has emerged as a substantial safety concern. Consequently, extensive research endeavors in recent years have been directed to the development of solid-state or quasi-solid-state electrolytes, aiming to replace the potentially hazardous flammable organic electrolytes. In pursuit of this objective, Segalman et al. introduced a groundbreaking approach by incorporating zwitterionic solid polymeric electrolytes (SPEs) featuring self-assembling superionically conductive domains.^17^ This unconventional design challenged traditional assumptions that ordered domains hinder ion mobility in SPEs. The integration of precisely tailored crystalline domains led to exceptional lithium conductivity and selectivity, effectively integrating the strengths of inorganic electrolytes with those of organic polymers to optimize the overall properties of SPEs.

The utilization of highly concentrated water-in-salt (WIS) electrolytes offers a promising avenue for the development of high-voltage aqueous batteries with improved safety. However, the physicochemical characteristics of WIS electrolytes are different from those of dilute electrolytes. In the investigation by Ge, Chen-Wiegart et al.,^18^ a comprehensive approach combining Raman tomography, operando X-ray diffraction refinement, and synchrotron X-ray 3D spectroscopic imaging is employed to investigate the chemical heterogeneity in LiV_3_O_8_-LiMn_2_O_4_ batteries utilizing WIS electrolytes and thick porous electrodes. The findings consistently highlight ionic diffusion in the electrolyte as the primary bottleneck impeding the performance of thick porous WIS batteries.

Machine learning has found broad application in accelerating the discovery of diverse functional materials for energy storage. Wang, Choy, and co-workers effectively utilized machine learning to analyze historical experimental data to predict initial and cycle discharge capacities for various doped nickel cobalt manganese (NCM) materials employing nonlinear algorithms.^19^ This approach guided the estimation of properties in singly doped NCM materials, showcasing the potential of machine learning in optimizing the understanding and prediction of material behaviors in battery technologies. Gomez-Bombarell, Shao-Horn et al. employed a chemistry-informed machine learning model to precisely predict ionic conductivity in SPEs, incorporating the Arrhenius equation.^20^ This innovative model not only accurately identified potential SPE formulations but also revealed correlations between anion properties and predicted ionic conductivity. The imperative need for the integration of artificial intelligence tools, including machine learning and extensive data analysis, in the exploration of electrode materials, electrolytes, and battery management cannot be overstated.

## Energy Storage beyond the Lithium System

Due to the Earth’s abundant supply of sodium, sodium-ion batteries (SIBs) are emerging as a promising energy storage alternative to LIBs in terms of cost-effectiveness and sustainability. However, the operation of electrodes at potentials beyond their thermodynamic equilibrium presents challenges, particularly in the unstable interfaces of anode materials (such as hard carbons and sodium metals) with electrolytes, hindering the development of high-performance SIBs. In an Outlook presented by Zhou, Lu, and co-workers,^21^ the nanoconfining mechanism, aimed at manipulating the desolvation process, was extensively discussed as a strategy to address these challenges and enable the practical implementation of SIBs and anode-free batteries. The Outlook also provides guidelines for designing improved electrolytes and constructing stable interphases for SIBs.

In an Outlook by Yu, Chou et al., the research advances in the development of cathode materials for SIBs are summarized.^22^ The materials are classified into three different categories: transition metal oxides, polyanionic compounds, and Prussian blue analogs. Both the advantages and problems for each category are discussed in this Outlook. The authors also share their perspectives on the future development of SIB’s cathode materials. An Outlook by Zhao and co-workers discusses the progress of polyanion-type cathode materials, including V-based, Fe-based, and Mn-based polyanionic phosphate/sulfate compounds.^23^ While the synthesis of large-sized and dense particles of polyanionic cathode materials is needed to improve the volume energy density, there is a pressing need to enhance their intrinsic electron conductivity. Challenges are still ahead to achieve both high energy density and long cycling life for large-scale energy storage applications.

In the study by Wang, Ding et al.,^24^ they illustrate the important contribution of the *in situ* formation of the gel electrolyte, resulting from the interaction between the liquid electrolyte and a Li-modified Na anode, to the fabrication of stable rechargeable Na–air batteries. This gel formation retards the crossover of H_2_O and O_2_, delaying Na anode corrosion and electrolyte decomposition. The electrostatic shield effect of Li^+^ from the modified Li layer helps to suppress Na dendrite formation. As a result, the constructed battery exhibits excellent cycle performance over 2000 h in air. Moreover, the work by Yu et al. reports a proof-of-concept molten sodium battery operating at a low temperature of 100 °C using fusible Bi–Pb–Sn alloy as the cathode to inhibit metal dendrite growth.^25^ They propose the potential of these batteries for grid-scale energy storage.

Alongside SIBs, magnesium-ion batteries (MIBs) stand out as another alternative to LIBs due to their divalent charge of magnesium. Nevertheless, their progress is hindered by the scarcity of electrolyte systems capable of providing sufficient conductivity, low nucleophilicity, and chemical stability. Guo, Lavallo et al. demonstrate that the alkylation of carborane anions on their C-vertex helps to create Mg^2+^ electrolytes with improved solubility in a wide temperature window as well as high ionic conductivity and oxidative stability in 1,2-dimethoxyethane for better-performance MIBs.^26^

Due to the affordability of Fe and its salts, Fe metal batteries boast promising potential as long-lasting energy storage solutions for grid applications. However, the undesirable hydrogen evolution reaction (HER) often hampers the Coulombic efficiency of conventional Fe batteries utilizing aqueous electrolytes containing simple Fe salts. In a recent work by Bedrov, Gao et al., a breakthrough was achieved through the development of a series of iron electrolytes.^27^ By incorporating MgCl_2_ or CaCl_2_ into the FeCl_2_ electrolyte, they successfully enhanced the deposition/stripping efficiency to an impressive 99.1%. The remarkable improvement was attributed to the reduction in “dead Fe” and the suppression of HER. In classical aqueous redox flow batteries operating at a single pH value, cell potentials are often low. To enhance the potentials, Mallouk et al. demonstrated the use of bipolar membranes to enable positive and negative electrodes to operate in electrolytes of different pH values, one alkaline and the other acidic.^28^ The developed approach resulted in an open circuit voltage of approximately 1.6 V. To prevent capacity decay in vanadium redox flow batteries, Fan, Zhao et al. develop a valence regulation strategy aimed at preventing V^2+^ crossover by employing electrolytes with elevated average valence (V^3.68+^).^29^ This approach enhances the accumulated discharge capacity in 400 cycles by 52.33% compared to conventional electrolytes with V^3.50+^.

The swift evolution of transparent, flexible, and degradable electronic devices is driving the development of batteries with similar physical properties. An Outlook by the Niederberger group summarizes some research advancements in the development of batteries that are flexible, stretchable, transparent, and degradable.^30^ They focus on the challenges inherent in the battery architecture, covering aspects such as active materials, current collectors, electrolyte/separators, and packaging necessary for producing such batteries. To make flexible supercapacitors, Fang and colleagues developed a series of porous coordination cage materials with tunable M_4_-O cluster nodes and linker ligands. Among the developed materials,^31^ a Mn-based porous coordination cage material exhibits a molecular capacitance up to 2514 F mmol^–1^ and a high areal capacitance of 250 mF cm^–2^. The inner and external cavities of the material contribute to the pseudocapacitance and the electrical double-layer capacitance, respectively. The work by Hao co-workers reports the fabrication of asymmetric, flexible, and all-solid-state microsupercapacitors using hollow supraparticles composed of thousands of close-packed upconverting nanoparticles (UCNPs) via an emulsion assembly approach.^32^ After carbonization, the supraparticles are coated with polypyrrole to serve as the cathode material with a high gravimetric capacitance of 308.6 F g^–1^ at 0.6 A g^–1^. Integration of the supraparticles-based cathode with a silver current collector, activated carbon anode, and gel electrolyte, onto a PET substrate yields flexible microsupercapacitors capable of integrated energy conversion and storage.

## Electrocatalysts for the Interconversion of Electrical Energy and Chemical Energy

Electrocatalytic reactions provide an efficient method for transforming renewable electricity into chemical energy as well as converting chemical fuels into electricity. Much of the ongoing research in this field concentrates on the creation of electrocatalysts that are not only cost-effective but also exhibit high activity, selectivity, and stability. The advancement of electrocatalysts with precisely defined molecular structures is crucial for conducting in-depth structure-properties correlation studies. These investigations are essential for formulating specific design guidelines, thereby improving the development of superior electrocatalysts customized for various chemical reactions. Without a doubt, molecular catalysts supported on electronic conductors are an ideal class of electrocatalysts for investigating the electrocatalytic mechanism at the atomic level. For instance, Hammes-Schiffer and co-workers carried out density functional theory calculations to simulate π-stacked interactions or axial ligation to a surface oxygenate using both cluster and periodic models. Results show that concerted proton-coupled electron transfer to a graphite adsorbed cobalt tetraphenylporphyrin occurs with band to bond electron redistribution to drive HER in alkaline media. This insight has broad implications for the study of surface immobilized catalysts.^33^

Owing to their well-defined structures, metal–organic frameworks (MOFs) represent an ideal class of heterogeneous catalysts for exploring electrocatalytic mechanisms. As illustrated by Dincǎ, Unwin et al.,^34^ when supported on a gas diffusion electrode, the 2D conductive MOF, Ni_3_(HITP)_2_, displayed an outstanding activity in the oxygen reduction reaction (ORR), surpassing rates in the H-cell by over 100-fold for ORR activity and more than 740-fold for H_2_O_2_ electrosynthesis. Leveraging scanning electrochemical cell microscopy to optimize mass transport, the ORR activity was further improved to 1200 mA cm^–2^. These results highlight the critical role of mass transport control in molecular materials, emphasizing its significance in achieving high-current-density electrocatalysis. In their Outlook, Liao, Chen, and co-workers highlighted the clear, designable, and tunable catalytic active sites and chemical microenvironments of MOF materials for investigating structure–performance relationship in the CO_2_ reduction reaction (CO_2_RR).^35^ Recent advances of using MOFs as CO_2_RR catalysts were summarized to explore the impact of various factors of MOFs on their CO_2_RR performance. These factors include the metal sites, metal coordination geometry, electronic structure of the active site, the secondary coordination sphere or microenvironment, the strength of coordination bonds, stability of organic ligands, ligand conductivity, and the nature of the electrolytes. Together with rational design strategies of MOF materials, the electrode fabrication and electrolyzer design to enhance the CO_2_RR performance of MOF electrocatalysts were also discussed.

Cu catalysts have been well documented in the literature as excellent electrocatalysts for CO_2_RR to minimize H_2_ production for the preferential production of different organic compounds. Zhang, Zheng et al. showcased the modification of Cu catalysts using amine-containing dendrimers to tailor microenvironments, promoting the preferential production of acetate.^36^ This selectivity was facilitated by the modified environment, which increased the CO coverage on Cu sites and raised the pH value, favoring the formation of the ethenone intermediate. Through the amine-modification method, one of the highest CO_2_-to-acetate Faradaic efficiencies of 47.0% was achieved with a partial current density of 202 mA cm^–2^ at −0.97 V versus RHE.

## Electrochemical Conversion beyond Electrocatalysts

In addition to electrocatalysts, ion-exchange membranes represent another pivotal material for constructing devices for efficient electrochemical reactions. While proton-exchange membranes (PEMs) have been extensively developed for applications such as PEM fuel cells and water electrolyzers, there has been a growing focus on the advancement of durable, high-performance anion-exchange membranes (AEMs). This attention stems from the potential to fabricate high-performance electrochemical devices without relying on costly precious metals. An Outlook presented by Xu, Ge, and colleagues provides a comprehensive summary of the recent research strides made in AEMs featuring diverse polymeric backbone structures.^37^ They delve into the synthesis methodologies employed and explore the intricate relationship between membrane properties, such as conductivity, chemical robustness, and mechanical integrity, and the underlying polymer backbone. Moreover, the Outlook offers valuable insights into future research directions aimed at refining the structural design of AEMs, with the overarching goal of realizing durable electrochemical devices.

Besides essential component materials, meticulous device design plays a crucial role in optimizing efficiency in electrochemical reactions. For instance, the research conducted by Berlinguette and colleagues underscores the significance of electrolyzer design in enhancing both energy efficiencies and CO_2_ utilization efficiencies in CO_2_RR.^38^ A so-called “bicarbonate electrolyzer” was designed by the research team to enhance the CO_2_RR performance. This innovative design involves reducing a KHCO_3_-enriched reactive carbon solution rather than gaseous CO_2_ at the cathode, conducting the hydrogen oxidation reaction (HOR) at the anode and incorporating a cation exchange membrane in lieu of an anion exchange membrane between them. This configuration allows for a remarkable CO_2_ utilization efficiency of 40%, achieving a partial current density for CO of up to 220 mA cm^–2^ at a voltage of 2.3 V. Importantly, such an electrolyzer design facilitates the coupling of CO_2_RR with green H_2_ production.

Electrochemical reactions offer an effective pathway for synthesizing chemicals that are challenging to produce through traditional methods. For example, the electrochemical reduction of phosphate salts enables the sustainable production of elemental white phosphorus (P_4_); however, this requires the cleavage of strong and inert P–O bonds. Surendranath and his colleagues have shown that the low oxide anion activity, characterized by Lux-Flood acidity, effectively promotes the activation of P–O bonds in molten sodium trimetaphosphate.^39^ The Lux acidic phosphoryl anhydride linkages play a crucial role in facilitating the selective and efficient electrosynthesis of P_4_, achieving an impressive 95% Faradaic efficiency. These findings lay the groundwork for potentially developing environmentally friendly alternatives with lower carbon impact compared to traditional carbothermal synthesis methods for P_4_.

Electrochemical reactions can also help to create effective methods for CO_2_ separation from the atmosphere using electricity rather than thermal energy. Gallant et al. demonstrate a dual salt cation-swing process to electrochemically modulate the CO_2_ loading on liquid amine sorbents in dimethyl sulfoxide.^40^ A prototype electrochemical cell was built by using Prussian white as the K^+^ intercalation cathode, and Zn foil as the anode in an ethoxyethylamine/DMSO electrolyte containing a dual KTFSI/Zn(TFSI)_2_ salt. During discharging, the dual-ion cell intercalates K^+^ into the Prussian white cathode and strips Zn^2+^ from the Zn anode. The reaction of Zn^2+^ with carbamic acid (RNHCOOH) forms carbamate (RNHCOO^–^), leading to the release of CO_2_ caused by proton transfer. During charging, while K^+^ is released from the cathode, Zn^2+^ is deposited back to the Zn anode. With proton transfer from amine to carbamate forming carbamic acid, CO_2_ can be recaptured by the amine. A low CO_2_ separation energy of ∼22–39 kJ/mol CO_2_ was demonstrated.

To meet the demands of real commercial applications, both the performance and durability of electrochemical devices are critical. Under the operational conditions of electrochemical conversion systems, the real performance of electrocatalysts highly depends on their surrounding microenvironment. For example, the electrocatalysts in the catalyst layer must be blended with ionic-conducting ionomers to build a conductive porous assembly to ensure the good transport of reactants, products, ionic species and also electrons. The assembly structures are dynamic under the electrochemical reactions. In order to have high durability in performance, the development of strategies to stabilize the catalyst layer’s assembly structure is important. As demonstrated in alkaline membrane fuel cells and water electrolyzers, Lee and co-workers developed an *in situ* cross-linking strategy to strengthen the interactions within the catalyst layer and also the interactions between the catalyst layer and anion exchange membrane (AEM).^41^ In the developed strategy, alkyne motifs were grafted onto the backbones of the ionomer and AEM, triptycene branched poly(fluorenyl-*co*-biphenyl *N*-methylpiperidine) (Trip-PFBM) and poly(dibenzyl-*co*-terphenyl *N*-methylpiperidine) (PDTM), respectively. After the cross-linking treatment with heating at 170 °C under a vacuum, the catalyst layers in AEM fuel cells and electrolyzers exhibited much enhanced performance and durability. A high current density up to 15.17 A cm^–2^ at 2.0 V was even achieved by an AEM electrolyzer using the cross-linked catalyst layer. Zhang, Zheng, and co-workers developed a phosphonated ionomer featuring an intrinsic microporous framework and durable proton-conductive −PO_3_H_2_ groups.^42^ As a high-performance catalyst binder, the ionomer helps to construct stable mass transport pathways to enhance the electrode performance for high-temperature PEM fuel cells. At the system level, a single-cell PEM water electrolyzer is assembled with a PEM membrane sandwiched by catalyst layers, porous transport layers, and flow channel plates. Tao, Wang et al. demonstrate that the structure of flow channels readily influences of the stress applied on the catalyst layer.^43^ Using a mesh flow channel with gradient pores can reduce the stress inhomogeneity on the catalyst layer. The optimized stress distribution helps to enhance the in-plane transport of electrons for achieving high performance and durability.

Although the successful demonstration of large-scale green hydrogen production using renewable electricity has been achieved, hydrogen storage is still a big challenge for many applications of green hydrogen. Beller, Junge, and co-workers demonstrate a hydrogen storage and release process through the catalytic interconversion of (bi)carbonate and formate salts in the presence of manganese pincer catalysts and glutamic acid.^44^

This collection links energy generation, storage, and use with the principles of a circular carbon economy, highlighting the multifaceted nature of the energy landscape. The development of renewable energy systems and a green society requires joint efforts from both academic and industrial communities. The advancement of high-performance materials is crucial; however, their compatibility with complementary components is equally vital. This synergy enables the creation of durable, cost-efficient devices with enhanced capabilities. While numerous promising materials and innovative concepts have been demonstrated for various energy-related processes, overcoming several hurdles is necessary to implement them in real-world applications. We anticipate that readers will gain valuable insights into the circular green energy economy from this collection, prompting exploration into diverse avenues for future research. By striving toward the development of carbon-zero renewable energy technologies, we aim to foster tangible benefits for our society, paving the way for a sustainable and prosperous future.
